# Biodiversity response to forest management intensity, carbon stocks and net primary production in temperate montane forests

**DOI:** 10.1038/s41598-020-80499-4

**Published:** 2021-01-15

**Authors:** Thomas Asbeck, Francesco Sabatini, Andrey L. D. Augustynczik, Marco Basile, Jan Helbach, Marlotte Jonker, Anna Knuff, Jürgen Bauhus

**Affiliations:** 1 Chair of Silviculture, University of Freiburg, Tennenbacher Str. 4, 79106 Freiburg, Germany; 2 German Centre for Integrative Biodiversity Research (iDiv) - Halle-Jena-Leipzig, Puschstraße 4, 04103 Leipzig, Germany; 3 Martin-Luther-Universität Halle-Wittenberg, Institut Für Biologie, Am Kirchtor 1, 06108 Halle, Germany; 4 Chair of Forestry Economy and Forest Planning, University of Freiburg, Tennenbacher Str. 4, 79106 Freiburg, Germany; 5 Chair of Wildlife Ecology and Management, University of Freiburg, Tennenbacher Str. 4, 79106 Freiburg, Germany; 6 Chair of Geobotany, University of Freiburg, Schänzlestrasse 1, 79104 Freiburg, Germany; 7 Forest Research Institute of Baden-Württemberg (FVA), Wonnhaldestraße 4, 79100 Freiburg, Germany; 8 Chair of Nature Conservation and Landscape Ecology, University of Freiburg, Tennenbacher Str. 4, 79106 Freiburg, Germany

**Keywords:** Forestry, Ecosystem ecology

## Abstract

Managed forests are a key component of strategies aimed at tackling the climate and biodiversity crises. Tapping this potential requires a better understanding of the complex, simultaneous effects of forest management on biodiversity, carbon stocks and productivity. Here, we used data of 135 one-hectare plots from southwestern Germany to disentangle the relative influence of gradients of management intensity, carbon stocks and forest productivity on different components of forest biodiversity (birds, bats, insects, plants) and tree-related microhabitats. We tested whether the composition of taxonomic groups varies gradually or abruptly along these gradients. The richness of taxonomic groups was rather insensitive to management intensity, carbon stocks and forest productivity. Despite the low explanatory power of the main predictor variables, forest management had the greatest relative influence on richness of insects and tree-related microhabitats, while carbon stocks influenced richness of bats, birds, vascular plants and pooled taxa. Species composition changed relatively abruptly along the management intensity gradient, while changes along carbon and productivity gradients were more gradual. We conclude that moderate increases in forest management intensity and carbon stocks, within the range of variation observed in our study system, might be compatible with biodiversity and climate mitigation objectives in managed forests.

## Introduction

Forests are at the heart of the debate around two major societal challenges: climate change and the biodiversity crisis^1^. European forests harbor a large share of the continent’s biodiversity and make an important contribution to climate mitigation^2,3^, despite the fact that previous land-use changes and a long tradition of management has altered their structure and composition substantially^4^. They are also increasingly affected by climate change, which might threaten the provisioning of important ecosystem services as well as forests’ support for biodiversity^5,6^. Increasing temperatures, coupled with changes in precipitation and disturbances such as droughts and storms are already pervasively altering vegetation dynamics^7^. By manipulating forest structure and composition, management can play an important role in developing adaptive solutions to secure the long-term delivery of forests’ ecosystem services, while contributing to halting biodiversity loss and mitigating climate change^8–12^.

Through changes in the composition, structure and spatial arrangement of trees, management can have profound effects on forest ecosystems^13^. By extracting timber, management affects forest carbon stocks and provides a renewable biomaterial for substituting fossil energy and energy-intensive products^2^. Harvesting, in turn, releases resources and growing space, which ultimately influence forest productivity. Furthermore, management changes the availability and recruitment rate of deadwood, which is an important substrate for many forest organisms^14^. Finally, by selectively harvesting larger and older trees, management might reduce the availability of microhabitats^15^ with direct and indirect effects on biodiversity^16^. As such, forest management plays a key role in determining both the amount of habitat available to support biodiversity as well as the stocks of carbon in different ecosystem pools. Hence, there is a great interest in developing management approaches that simultaneously support biodiversity, provide wood products, and optimize climate mitigation benefits. Yet, the actions needed to halt biodiversity loss may differ from those required to mitigate climate change^17^. It is unclear, for instance, to what extent practices such as retention forestry^18^ or cessation of management^19^ could increase in situ carbon storage and benefit biodiversity alike^20,21^. Thus, the question of trade-offs between forest management, carbon storage and maintenance of productivity in relation to forest biodiversity has recently gained attention^22–26^.

The direct and indirect effects of forest management on biodiversity are not fully understood^24^. This is possibly due to the inherent difficulty of summarizing management regimes into clearly distinguishable entities^27^. The use of simplified classes describing complex silvicultural systems frequently delivers an overly crude representation of management effects^25,28^. For instance, a mere distinction between uneven- and even-aged silvicultural systems may lack the nuance needed to understand the complex relationships among management, structure, productivity and biodiversity in forests^29^. Indeed forest structures, and the depending biodiversity, correlate only weakly with management categories^30–32^. To overcome this problem, Kahl and Bauhus (2014) proposed to quantify forest management as a gradient of intensity based on three aspects: (a) the appropriation of woody biomass measured as the proportion of harvested tree volume compared to the theoretical maximum stocking volume, (b) the change in tree species composition measured as the proportion of non-native vs. native trees in a stand, and (c) the maintenance of natural stand structural dynamics measured as the proportion of dead wood originating from harvesting activities (e.g., stumps and crown wood) vs. that of natural origin. Avoiding the use of strict categories, this approach allows the quantification of management intensity as a continuous variable and accounts for the multifaceted influences of forest management. This approach has proved useful when assessing the influence of forest management intensity on biodiversity (e.g. Seibold et al.^34^).

Complexity is ubiquitous in biological systems. Yet, research exploring the relationships between forest management, carbon stocks, productivity and biodiversity often relies on simplified considerations of these aspects. For instance, when considering the influence of carbon stocks on biodiversity, most studies only focused on above-ground carbon stocks^22,35^. Much less attention has been given to the contribution of belowground, root and soil organic carbon stocks, possibly because detailed data on these carbon pools is rarely available^36^. Here we used aboveground carbon in wood and foliage as well as belowground root carbon stocks as predictor variables. Similarly, the biodiversity-productivity relationship has often been explored in relation to tree species composition only^24,37^, while studies on the influence of net primary productivity on forest-dwelling species returned unclear results^23,38^. Consequently, there is limited knowledge on the relationship between richness and composition of different taxonomic groups and forest productivity. Yet, understanding how the different components of management intensity, carbon pools and productivity collectively shape the diversity of different taxonomic groups is crucial to inform forest managers on how to reconcile different management objectives.

The exact shape of the response of biological assemblages, here referring to species composition or structural indicators such as tree-related microhabitats, to changes in forest structure is also uncertain. A key question is whether changes in species assemblages are gradual or abrupt along gradients of management intensity, carbon stocks and productivity. It is similarly unknown whether these changes are similar across taxonomical groups in temperate forest^39^, since most previous research on ecological thresholds focuses either on single species or different ecosystems^22,40^. Recent evidence suggests that ecological thresholds may exist, i.e. transition points of relatively rapid change between different ecosystem states or ecological conditions in response to small, continuous changes in one causal variable^39^. Identifying these thresholds would help understand which facet of management has the strongest leverage on species turnover, and define ‘safe operating spaces’ for manipulating forest carbon or productivity without triggering undesired changes in biological communities^41^. This could provide relevant and timely information for forest managers, and support the establishment of quantitative criteria to safeguard biodiversity in managed forests^42^.

Here, we use forest and biodiversity data from a large-scale collaborative project in southwestern Germany^43^ to disentangle the relative influence of forest management intensity, carbon stocks and forest productivity on forest biodiversity. Our study was based on information from 135 one-hectare forest plots comprising a high level of detail on past management intensity, tree biomass carbon stocks (three pools: aboveground wood, roots and foliage), forest productivity, and richness of a broad range of forest-dwelling taxonomic groups including birds, bats, insects, and vascular plants, and tree-related microhabitats (here considered as a structural indicator of biodiversity). Specifically, we tried to answer three questions:

1. What is the relative influence of forest management intensity, tree biomass carbon stocks and productivity in determining forest biodiversity across multiple taxonomic groups?
2. Which facet of forest management intensity, and which carbon pool is most strongly related to species richness of different taxonomic groups?
3. Do biological assemblages, here the composition of species groups and tree-related microhabitats, vary gradually or abruptly along gradients of forest management intensity, carbon stocks and productivity?

## Results

We found a considerable species richness across taxonomic groups, even if the variability across plots was relatively low (see Table S1). Specifically, we found a maximum richness of 31 bird species, 11 acoustic groups of bats, 71 vascular plant species, 18 insect orders, and 9 types of tree-related microhabitats per plot. The maximum pooled species richness was 0.86 (i.e., the average species richness across taxonomic groups, scaled by the total number of species found for each group, with the exclusion of tree-related microhabitats). The mean pooled species richness was 0.67 with a standard deviation of 0.08. Forest management intensity and carbon stocks varied within the expected limits of mature temperate forests across plots (Table 1). For productivity, which we assessed using the normalized difference vegetation index (NDVI) based Sentinel 2A images^44,45^ as a proxy, the plot-to-plot variability was much lower (Table 1). This low variability in productivity is not surprising since all plots are located in mature, mixed mountain forests, which are managed using continuous cover systems^43^. Forest management intensity, total carbon stock and productivity were not correlated, although the individual components of these three main variables were, at least to some extent related, which was expected (Table S2).

**Table 1:** Summary statistics of the variables used in the statistical analyses across 135 one-hectare forest plots.

Variable	Minimum	Maximum	Mean (SD)
Forest management intensity	0	2.4	1.2 (0.51)
Ratio of deadwood volume of man-made vs. natural origin (Idwcut)	0	1	0.4 (0.28)
Ratio of volume in non-native vs. native tree species (Inonat)	0	0.9	0.4 (0.29)
Ratio of harvested tree volume vs. maximum total stand volume (Iharv)	0	0.8	0.4 (0.17)
Total tree biomass carbon (Mg C ha^−1^)	48.5	388.4	184.3 (55.0)
Aboveground carbon in wood (Mg C ha^−1^)	28.9	228.6	(33.2)
Foliage carbon (Mg C ha^−1^)	0.8	15.7	(2.5)
Root carbon (Mg C ha^−1^)	19.6	159.9	72.6 (23.0)
Normalized difference vegetation index (NDVI)	0.6	0.8	0.7 (0.04)

### Weak effects of management intensity, carbon and productivity on forest diversity

**Figure 1:**
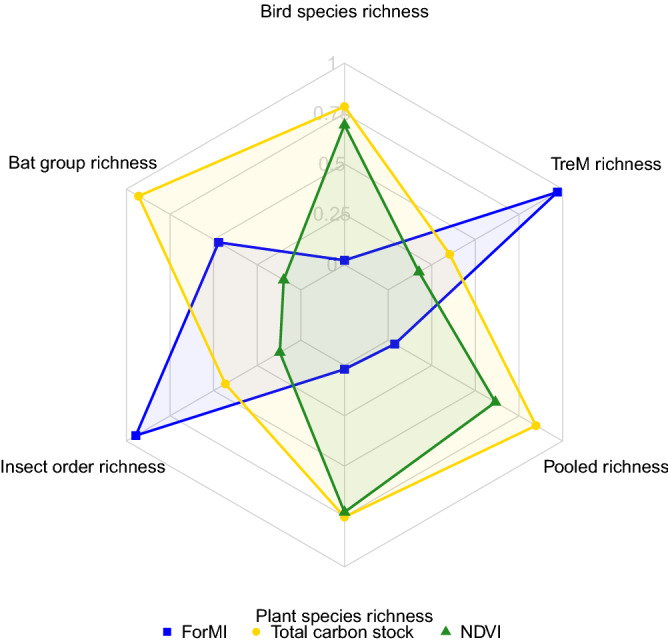
Scaled relative importance of forest management intensity (ForMI), total tree biomass carbon stock and NDVI on the richness of four taxonomic groups, the pooled richness of those groups and richness of tree-related microhabitats. Relative importance ranges between 0 and 1. This graph was produced in R using the *fmsb* package^46^.

Forest management intensity, total biomass carbon stock and productivity were weak predictors of the total pooled species richness across different taxonomic groups, based on the output of machine-learning non-parametric models (Pseudo R^2^ = − 0.16, see Table S3). Considering the different taxonomic groups separately did not improve predictions substantially (Pseudo R^2^ range from − 0.16 to − 0.03, see Table S3). Even if the overall influence was weak, management intensity was the predictor with the largest relative importance on insects and tree microhabitats (Fig. 1), while total carbon stock was the most important predictor of bat, bird, vascular plant and pooled species richness (Fig. 1). Net primary productivity, expressed as the NDVI, was never the variable having the highest importance for any of the taxonomic groups, although for plants its relative importance was comparable to that of carbon stock (Fig. 1).

### Disentangling the role of different components of forest management intensity and different carbon pools on taxonomic groups

**Figure 2:**
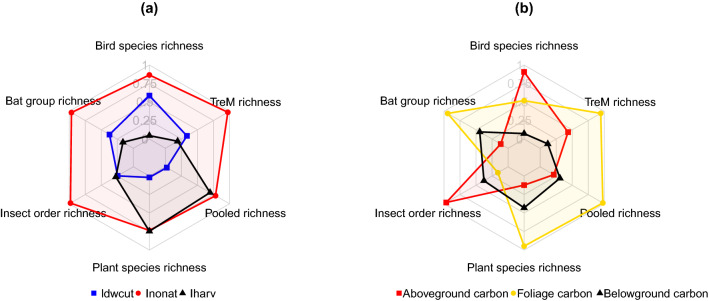
Scaled relative importance of the three parts of the a) forest management intensity (Idwcut: volume ratio of harvesting-related deadwood vs. deadwood of natural origin, Inonat: Ratio of volume in non-native vs. native tree species , Iharv: Ratio of harvested tree volume vs. maximum total stand volume) and b) three carbon pools (aboveground, foliage, and belowground) on the richness of four taxonomic groups, the pooled richness of those groups and richness of tree-related microhabitats. Relative importance ranges between 0 and 1. This graph was produced with R software and the *fmsb* package^46^.

Even considering the different components of forest management intensity and the different carbon pools separately did not return better models of the richness of different taxonomic groups (Pseudo-R^2^ range: − 0.23 to − 0.05, Table S3). Decomposing forest management intensity into the three components as suggested by Kahl and Bauhus (2014) revealed a relatively high importance of the share of tree species not native to the site, in this study mostly Norway spruce (*Picea abies*), on most taxonomic groups except plants (Fig. 2a). Only for vascular plants, harvesting intensity had a slightly higher importance (Fig. 2a) than the share of non-native tree species.

Disaggregating total tree biomass carbon stocks into three components (i.e., aboveground, root and foliage pools), revealed that aboveground carbon pool had the greatest relative importance for birds and insects, and foliage carbon had the highest influence for the remaining groups (Fig. 2b).

### Biological assemblage variation along gradients of forest management intensity, carbon stocks and productivity

**Figure 3:**
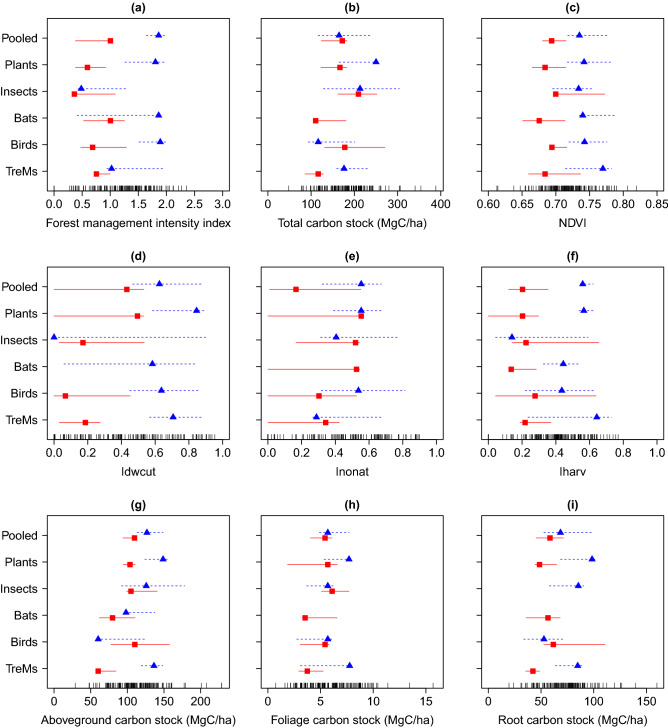
Change points of the aggregated sum(z−) and sum(z+) values for pooled richness, the species groups and tree-related microhabitats. These change points indicate where, along the gradients, species increase (positive, blue triangle) or decrease (negative change point, red square) their frequency. If symbols are missing, the respective group did not show a reliable negative or positive change along the gradient. Evidence for community-level thresholds among negative and positive taxa is assessed separately by tabulating and summing all negative change points (z−) and positive change points (z+) scores^39^. Sharp, nonlinear responses in taxon richness are reflected by relatively narrow intervals between upper and lower change point quantiles (5%, 95%), indicated by shorter whiskers. In cases where a species group shows a linear or more gradual response, they have broad whiskers spanning most of the range of the predictor. This graph was produced in R using the *TITAN2* package^39^.

The analyses of thresholds along the indicator gradients did not show fully congruent patterns across separate and pooled taxonomic groups and tree-related microhabitats (Fig. 3). The pooled species composition changed in a relatively abrupt way in relation to increasing forest management intensity (Fig. 3a). When pooling taxonomic groups, we found a substantial turnover in species composition at a value of management intensity of 1.9 (see Table S4). Above this level, sensitive species were replaced by management-tolerant species (Fig. 3a). The composition of plant and bird groups closely mirrored this pattern.

Disaggregating forest management intensity into its three components revealed that there was no abrupt change in species composition of taxonomic groups with the share of man-made vs. natural deadwood or the share of non-natural tree species, despite tree-related microhabitats indicating a possible abrupt change, which however did not reach the threshold level (Fig. 3e, Table S4). Conversely, we found clear thresholds for the composition of plant (0.6) and pooled species groups (0.6) when the ratio of harvested wood volume (Iharv) increased beyond these values (Fig. 3f, see Table S4).

We could not find detectable thresholds in species composition along the gradient of total carbon stocks for any of the taxonomic groups, except tree-related microhabitats (116.5 Mg C ha^−1^) (Fig. 3b, see Table S4). Yet, the composition of tree-related microhabitats shifted relatively abruptly when aboveground carbon stocks reached 60.2 and 136.3 Mg C ha^−1^. We detected an abrupt shift in species composition of plants and pooled taxonomic groups, for aboveground carbon levels of 109.7 and 103.6 Mg C ha^−1^, respectively (Fig. 3g). The composition of tree-related microhabitats changed abruptly when root carbon decreased below 42.3 Mg C ha^−1^. For plants, the threshold was 48.5 Mg C ha^−1^ (Fig. 3i). We found no clear thresholds in response to productivity for any of the groups considered (Fig. 3c).

## Discussion

In our study on temperate, mountain forests, we found a weak relationship between the richness of species or orders of different taxonomic groups and management intensity, tree biomass carbon stocks and productivity. Even if the main predictor variables had low explanatory power, management intensity was the most important predictor of tree-related microhabitats and insect order richness, whereas the richness of bats, birds and plants was mostly related to total carbon stocks. In addition, we found several clear thresholds for changes in the species composition in relation to forest management intensity, suggesting that management has an effect on forest communities, which goes beyond a mere change in the number of species or orders.

The weak overall effect of management intensity, carbon and productivity on the species or order richness of different taxonomic groups is in itself an important result. It clearly shows that factors other than those included here are more relevant for forest biodiversity, at least at the level of species richness. Evidence from the literature remains equivocal with studies that found species or order richness of different taxa to be affected^47^ by forest management intensity or to be independent from it^22,48^. There are also studies showing that forest harvesting intensity in southwestern Germany has little influence on structural diversity and hence on habitat diversity^13^. Only in the case of insects and tree-related microhabitats, forest management intensity had a higher relative importance than carbon stocks. The weak relationships between our predictor variables and biodiversity measures may be attributable to the fact that some of the most species-rich taxonomic groups in forests were either not included (fungi) or were only partially captures (flying insects) and determined at the level of orders and not species (arthropods). However, another study carried out in three regions in Germany also showed that forest management intensity had no influence on the decline in arthropod species numbers observed over a ten-year period from 2008 to 2017^34^.

Yet, comparing the predictive power of management, tree biomass carbon stocks and productivity showed that carbon stock had the highest influence on most taxonomic groups, including birds, bats and plants. Carbon stock is an important aspect of forest structure and has often been found important for biodiversity^24,32^. Carbon in tree foliage, in particular, had the strongest influence on the species or order richness of most groups, despite being the smallest among the carbon pools considered. This might reflect a difference between mixed broadleaf and purely coniferous stands in our study system^49^. In plots with a high basal area of broadleaves, the foliage carbon stock was lower compared to plots with a high basal area of conifers. Hence, foliage carbon pools might relate to resource availability, such as the amount of light available for ground-layer plants or the extent of the hunting range for bats.

Overall, species communities changed relatively abruptly when considering the gradient of forest management intensity, but only gradually with respect to forest carbon or productivity. Decomposing forest communities into taxonomic groups showed that this pattern might be related to an increase in bird, bat and plant species that are either tolerant to management or promoted by it. We hypothesize that higher forest management intensities correspond to stands having a more open canopy, which allows better foraging options for bats and more light availability for understory plants, as found in other studies^25,50,51^. Forest management might also affect bird species communities in mature managed forests, by increasing stand-level structural heterogeneity^13,27,52,53^, and creating suitable conditions for species that demand more open conditions (early successional specialists)^54^. A shift in composition of tree-related microhabitats appeared to occur in less intensively managed forests, especially those where the proportion of non-native tree species was lower, as tree species is a main driver of microhabitat richness^16^. This is in agreement with previous research showing a decrease in tree-related microhabitats in stands dominated by the introduced Douglas fir (*Pseudotsuga menziesii*)^55^.

Our results suggest that neither increasing nor decreasing tree biomass carbon stocks within the range of management currently observed in our study system will lead to abrupt changes in the composition of the inventoried groups. Species composition showed little sensitivity to changes in total carbon stock, as was found in previous studies in beech and oak dominated European forests^22^. Yet, in the case of tree-related microhabitats we did find a clear threshold at carbon stocks of ca. 120 Mg C ha^−1^. This suggests that large trees and their microhabitats should be maintained in managed areas^15,56,57^. Large trees provide a significantly higher variety of tree-related microhabitats compared to small trees^16,30^. Species composition in most taxonomic groups changed only gradually with forest productivity. The only exception were bats with a relatively stable species composition across the productivity gradient, which is in agreement with previous research^58^.

Even if based on a relative large sample, our study does not come without uncertainties and limitations. First, it only focused on temperate mountain forest ecosystems older than 60 years. All our study plots were located in state forests experiencing similar management regimes, therefore encompassing relatively short gradients in carbon stocks and productivity, at least compared to the overall regional variability. Additionally, the absence of early successional and old-growth forests probably reduced the range of species or order richness for most taxonomic groups. This might partly explain why we could not find strong relationships between species richness and productivity or carbon stocks, or clear shifts in community composition with measures of forest management intensity. Spatial scale might also have played a role. Our plots were one hectare in size, which is a standard size for sampling forest structure, but might be suboptimal to capture all bird^59^ or insect species^60,61^. In addition, the sampling methods for specific species groups do not provide a full census at the plot scale. For instance, we sampled only flying insects and flight interception traps might capture insects at different radii depending on the surrounding vegetation of the traps^62^. Finally, we only focused on a snap-shot of biodiversity conditions, which likely underestimates the temporal dimension of biodiversity variation along forest succession^27^.

## Conclusions

Several conclusions can be drawn from our study. First, the investigated richness of species and orders in different taxonomic groups was influenced only to a small extent by forest management intensity, tree biomass carbon stock and productivity in mature, temperate forests. Secondly, biodiversity-oriented forest management is compatible with the current range in carbon stocks and degrees of forest management intensity, which is in agreement with studies in other temperate forest types^17^. Hence, there is a high degree of flexibility in forest management on how to achieve biodiversity and climate mitigation objectives. For example, instead of further increasing carbon stocks in managed forests, more biomass carbon may be used to substitute fossil fuels and energy intensive materials^2^ without affecting the richness of forest dwelling species. This, however, only applies to the range of variation assessed here, since at higher carbon stocks, such as those encountered in old-growth forests, other specialized organisms might replace those commonly occurring in managed forests^63^. Our study further underlines the importance of analyzing the intensity of forest management, to balance the trade-offs detected in our results and increase habitat availability for multiple taxa across management systems^64^. Future studies could especially expand the intensity gradient by adding long-term unmanaged or primary forests as well as more intensively managed systems. In addition, the analyzed species groups may be expanded to include those that are more responsive to the diversity of forest developmental stages, e.g. fungi and coleoptera.

## Material and methods

We used observational multi-taxon and structural data collected within the project “Conservation of forest biodiversity in multiple-use landscapes of Central Europe—ConFoBi”^43^. Biodiversity data covers four taxonomic groups (bats, birds, insects and ground flora) as well as tree-related microhabitats. The data collection took place in 135 one-hectare forest plots located on state land in the Black Forest region (Latitude: 47.6°–48.3°N, Longitude: 7.7°–8.6°E, WGS 84). The plot selection followed a landscape gradient of forest cover in the 25 km^2^ surrounding the plots and a gradient of structural complexity indicated by the number of standing dead trees per plot. For details on plot selection, see Storch et al.^43^. All plots were located in stands older aged between 60 and 120 years, thus in mature forests. The majority of plots were managed for timber production; a few plots were in strict forest reserves (N = 7) that were set aside from management 20 to 40 years ago. The main tree species were Norway spruce (*Picea abies (L.)*) (share of 41%), European beech (*Fagus sylvatica (L.))* (22%) and silver fir (*Abies alba (Mill.)*) (19%). In each plot, we performed a forest inventory and measured the position, species, and diameter of all trees (live and dead) with diameter at breast height (DBH) larger than 7 cm.

### Inventory of tree-related microhabitats

The data of tree-related microhabitats were collected based on a detailed typology proposed by Larrieu et al.^65^. Tree-related microhabitats are defined as “a distinct, well delineated structure occurring on living or standing dead trees, that constitutes a particular and essential substrate or life site for species or species communities during at least a part of their life cycle to develop, feed, shelter or breed”^65^. Tree-related microhabitats correlate with bird, bat and (saproxylic) insect diversity^66,67^. These microhabitats include, for instance, woodpecker cavities, trunk and mould cavities, branch holes, dendrotelms, insect galleries, bore holes, stem injuries and wounds (e.g., bark loss, or exposed sapwood), crown deadwood, cankers and burrs, epiphytes, nests of vertebrates and invertebrates.

We recorded the position of all inventoried trees, their diameter at breast height (DBH), species and tree-related microhabitats in the snow and leaf-free period between fall 2016 and spring 2017. We used binoculars to identify microhabitats in the canopy. More detailed information on the data collection can be found in Asbeck et al.^30^.

### Biodiversity sampling

To infer about the biodiversity of managed forests in our research area, we sampled four taxonomic groups: insects, bats, birds and the ground-level vascular vegetation (referred to as plants). We used presence-absence data for the analyses.

We used window traps with collectors at the bottom and at the top of transparent plastic panes that served as flight barrier to sample flying insects in the forest understory^62^. Catches were removed on a four-weekly interval. Data on catches from mid-March to mid-July in 2017 were used for analysis. Arthropods were stored in 75% ethanol and sorted to order level (with Hemiptera being further separated into Auchenorrhyncha, Sternorrhyncha and Heteroptera). Larvae and non-flying taxa such as spiders were excluded from the dataset^68^.

During the summer 2016 and 2017 (May–October) bats sounds where recorded using Batloggers (Elekon AG, Lucerne, Switzerland). The ultrasonic sounds were analysed, identified, and manually verified using Batscope 3.2.0. We used the software Raven Pro 1.5.0^69^ for visual manual verification where needed. Undefined bat calls were omitted from the analyses.

Birds were sampled by employing standardized point counts with limited distance of 50 m, repeated three times during spring 2017 and 2018, starting half an hour after sunrise with the latest ending at 12:00 CET. A single count lasted 20 min and consisted of four 5-min-blocks, during which every bird heard or seen was recorded^67^.

To obtain species richness of the understory, all vascular plants below 5 m height were recorded on the 1 ha plots. We surveyed plants at the peak of the growing season, from August 2016 to July 2018.

Finally, we pooled species richness across taxonomic groups into a composite index (multi-diversity in Allan et al.^70^). We calculated pooled richness in a plot by averaging the species richness of different groups, each scaled by the total number of species found for that group.

### Forest management intensity index

To quantify the influence of management on biodiversity, we used the forest management intensity index (ForMI) as proposed by Kahl and Bauhus^33^. This index is composed of three components: (a) the proportion of harvested tree volume compared to the theoretical maximum standing tree volume (Iharv), (b) the proportion of volume in tree species not native to the site vs. stand volume in native trees (Inonat), and (c) the proportion of dead wood volume (e.g., crown wood) of anthropogenic vs. natural origin (Idwcut). To calculate components (a) and (b), we used data from the forest inventory and allometric functions. To calculate component (c), we combined the line intersect method, as described by Van Wagner^71^, to quantify volume of downed logs, with a 4 m wide strip transect to quantify the volume of tree stumps. We used a V-shaped transect, running from the North-East corner to the center of the southern plot border to the North-West corner. We measured the diameter of every piece of deadwood (> 7 cm) in five decay classes following established recommendations^72^, and recorded whether the origin was artificial or natural. All low stumps were assumed to stem from tree harvesting, in case this was not recognizable, since wind damage results either in uprooting or breakage of trees at higher stem sections. Heavily decayed log sections had to be traceable to uprooting or stem breakage to count as natural. Information on standing dead trees was obtained from the full stand inventory. One of the limitations of the forest management intensity index is that it can only assess forest management up to approximately 40 years in retrospect, since stumps of certain tree species are fully decayed within this period^33^.

### Carbon stocks

We used the forest inventory data and allometric relationships to quantify the carbon stocks of different compartments in each plot. Specifically, we estimated the carbon mass in foliage, stem, roots and aboveground carbon, assuming carbon accounted for 50% of the organic dry mass of all compartments. The allometric relationships for each species in the dataset were retrieved from Forrester et al.^73^. Equation (1) was applied to all trees included in the forest inventory and for all compartments. The stand scale results were obtained by aggregating the individual mass of all measured trees.

1 $$\ln (C_{is} ) = \ln (\beta_{0is} ) + \ln (\beta_{1is} DBH) + \varepsilon \quad forall i,\forall s$$

where: $C_{i}$ = compartment i of species s (foliage, stem, root and aboveground carbon mass); $\beta_{0i} ,\beta_{1i}$: coefficients for compartment i and species s; $DBH$: diameter at breast height (1.3 m).

### Forest productivity

Net primary productivity measures the carbon assimilated by plant photosynthesis net of autotrophic respiration. To quantify net primary productivity of forested areas, we used the normalized difference vegetation index (NDVI), as frequently done^44,45,74^. The relationship of the photosynthetic capacity of forest ecosystems has proven to be robust under a wide range of settings, including temperate forests^44^. We derived this data based on satellite images retrieved from Sentinel 2A on date August 23rd, 2016 for each plot. NDVI was calculated using the open-source software QGIS^75^.

### Statistical analyses

We used the random forests algorithm (*randomForest* package in R software^76,77^) to model the relationship between either the number of tree-related microhabitats or species richness (either pooled or considering different taxonomic groups separately), as a function of forest management intensity, total carbon stock and net primary productivity. We also ran additional (sub-)models relating each response variables to either the three components of the forest management intensity index, or to the three components of carbon stock. We used these models to quantify and compare the relative importance of different predictors.

The random forests algorithm uses decision trees, does not require prior assumptions and is less sensitive to collinearity among predictors compared to other statistical approaches^76^. Additionally, it flexibly models non-linear relationships, and allows to compare the relative importance of different predictors. We calculate the individual importance of each predictor by running 10.000 iterations. We set to three the number of variables randomly sampled as candidates at each split, since we included three predictors in each model. We then scaled the results to compare the relative importance of each predictor for the respective taxonomic group. We used the Pseudo r-squared returned by the random forest algorithm as a goodness-of-fit measure.

To test whether the composition of different taxonomic groups and tree-related microhabitats vary gradually or abruptly along gradients of forest management intensity, carbon and productivity, we used the threshold indicator taxa analysis (TITAN) as proposed by Baker and King^39^. This approach uses an indicator species analysis^78^ to optimally partition sample units along a continuous environmental gradient. To do so, TITAN differentiates between taxa increasing or decreasing in abundance along the environmental gradient, and returns cumulative z scores for negative [sum(z−)] and positive [sum(z+)] responses. By optimizing this z score, TITAN allows to identify the area of maximum aggregated change in the frequency and abundance of the considered taxa along the environmental gradient^79^. The performance of the indicator and the uncertainty around the location of change points are then estimated using bootstrapping.

We used TITAN to explore if species‐level change points aggregate to an abrupt threshold for the pooled species richness, and to check whether these change-points were congruent across taxonomic groups. In order to assess the variability across the taxonomic groups and tree-related microhabitats in community‐level change‐points, we ran TITAN both when considering all species together, and for each taxonomic group separately^22^. In line with previous applications of the method^50^, we only considered indicators having purity > 0.95 and reliability > 0.7. Purity quantifies the proportion of change-point response directions (positive or negative) among bootstrap replicates that agree with the observed response. Reliability indicates the proportion of bootstrap change points whose scores consistently result in P-values < 0.05. In few cases, we decreased the purity threshold of the indicators to a value of > 0.7 (Insects: forest management intensity index components, above- and belowground carbon; Bats: Idwcut, Inonat, above- and belowground; Birds: Inonat; tree-related microhabitats: aboveground carbon). We did this to provide the most complete overview of responses to the gradients, while still focusing only on robust trends. We considered an abrupt change whenever the whiskers of the change points did not cover more than 15% of the full gradient.

## Supplementary Information

Supplementary Tables.
