# Supplementary material for: Biodiversity response to forest management intensity, carbon stocks and net primary production in temperate montane forests
Source: Sci Rep. 2021 Jan 15;11:1625. doi: 10.1038/s41598-020-80499-4 (PMC7810709; doi:10.1038/s41598-020-80499-4)
Supplement: Supplementary file 1 — Supplementary Tables. [file 41598_2020_80499_MOESM1_ESM.docx]

# **Biodiversity response to forest management intensity, carbon stocks and net primary production in temperate montane forests**

Authors: Thomas Asbeck^1^*, Francesco Sabatini^2^, Andrey L. D. Augustynczik^3^, Marco Basile^4^, Jan Helbach^5^, Marlotte Jonker ^4,6^, Anna Knuff^7^, Jürgen Bauhus^1^

^1^ Chair of Silviculture, University of Freiburg, Tennenbacher Str. 4, 79106 Freiburg, Germany

^2^ German Centre for Integrative Biodiversity Research (iDiv) - Halle-Jena-Leipzig, Puschstraße 4, 04103 Leipzig, Germany; Martin-Luther-Universität Halle-Wittenberg, Institut für Biologie. Am Kirchtor 1, 06108 Halle. ORCID: <https://orcid.org/0000-0002-7202-7697>

^3^ Chair of Forestry Economy and Forest Planning, University of Freiburg, Tennenbacher Str. 4, 79106 Freiburg

^4^ Chair of Wildlife Ecology and Management, University of Freiburg, Tennenbacher Str. 4, 79106 Freiburg

^5^ Chair of Geobotany, University of Freiburg, Schänzlestrasse 1, 79104 Freiburg, Germany

^6^ Forest Research Institute of Baden-Württemberg (FVA), Wonnhaldestraße 4, 79100 Freiburg, Germany

^7^ Chair of Nature Conservation and Landscape Ecology, University of Freiburg, Tennenbacher Str. 4, 79106 Freiburg

*Corresponding author: Phone: 0761 2033675, [thomas.asbeck@waldbau.uni-freiburg.de](mailto:thomas.asbeck@waldbau.uni-freiburg.de), ORCID: https://orcid.org/0000-0003-4786-9312

# Supporting information

Table S1 – Species richness of bats, vascular plants, birds and order richness of insects, TreM and scaled pooled richness (excluding TreMs). Forest management intensity index (ForMI) with respective parts Idwcut (ratio of natural vs. man-made downed deadwood), Inonat (ratio of non-native vs. native tree species), Iharv (ratio of harvested vs. theoretical maximum standing volume). Total carbon stock with respective parts aboveground, foliage and root carbon. Normalized difference vegetation index (NDVI).

| Plot ID | ldwcut | Inonat | Iharv | ForMI | Total carbon stock (MgC/ha) | Aboveground carbon stock (MgC/ha) | Foliage carbon stock (MgC/ha) | Root carbon stock (MgC/ha) | NDVI | Bat richness | Bird richness | Plant richness | TreM richness | Insect richness | Scaled Pooled richness |
| --- | --- | --- | --- | --- | --- | --- | --- | --- | --- | --- | --- | --- | --- | --- | --- |
| 1 | 0.00 | 0.53 | 0.15 | 0.68 | 304.28 | 179.06 | 10.44 | 125.22 | 0.71 | 6 | 20 | 31 | 9 | 14 | 0.75 |
| 2 | 0.00 | 0.26 | 0.09 | 0.35 | 83.06 | 47.81 | 3.34 | 35.25 | 0.67 | 7 | 26 | 6 | 8 | 15 | 0.65 |
| 3 | 0.06 | 0.00 | 0.22 | 0.28 | 99.64 | 58.58 | 3.15 | 41.06 | 0.65 | 9 | 22 | 25 | 8 | 13 | 0.71 |
| 5 | 0.00 | 0.09 | 0.22 | 0.31 | 238.23 | 141.39 | 6.45 | 96.85 | 0.65 | 3 | 24 | 30 | 9 | 15 | 0.76 |
| 7 | 0.00 | 0.00 | 0.00 | 0.00 | 48.45 | 28.88 | 1.86 | 19.57 | 0.78 | 7 | 24 | 38 | 7 | 12 | 0.67 |
| 8 | 0.01 | 0.89 | 0.00 | 0.90 | 149.27 | 90.02 | 7.70 | 59.25 | 0.64 | 8 | 24 | 12 | 5 | 14 | 0.60 |
| 9 | 0.32 | 0.00 | 0.51 | 0.83 | 116.87 | 71.55 | 3.61 | 45.32 | 0.73 | 7 | 22 | 40 | 5 | 12 | 0.66 |
| 10 | 0.00 | 0.65 | 0.00 | 0.65 | 213.22 | 123.71 | 7.17 | 89.50 | 0.61 | 7 | 18 | 12 | 7 | 13 | 0.56 |
| 11 | 0.15 | 0.21 | 0.64 | 1.01 | 172.41 | 107.65 | 4.84 | 64.76 | 0.73 | 6 | 22 | 31 | 4 | 16 | 0.65 |
| 14 | 0.19 | 0.52 | 0.11 | 0.81 | 211.58 | 132.75 | 4.81 | 78.83 | 0.72 | 8 | 29 | 40 | 6 | 16 | 0.78 |
| 15 | 0.35 | 0.33 | 0.63 | 1.31 | 179.46 | 108.09 | 4.93 | 71.37 | 0.73 | 7 | 26 | 43 | 7 | 15 | 0.78 |
| 16 | 0.54 | 0.00 | 0.20 | 0.73 | 243.19 | 157.39 | 2.72 | 85.80 | 0.72 | 5 | 23 | 15 | 8 | 14 | 0.58 |
| 17 | 0.90 | 0.90 | 0.55 | 2.35 | 213.30 | 121.67 | 8.58 | 91.63 | 0.68 | 7 | 24 | 35 | 5 | 14 | 0.73 |
| 18 | 0.28 | 0.68 | 0.43 | 1.39 | 241.59 | 136.45 | 8.71 | 105.14 | 0.70 | 6 | 21 | 34 | 5 | 15 | 0.63 |
| 19 | 0.00 | 0.00 | 0.41 | 0.41 | 192.82 | 123.98 | 3.52 | 68.84 | 0.70 | 5 | 20 | 29 | 7 | 12 | 0.71 |
| 20 | 0.72 | 0.67 | 0.53 | 1.92 | 216.21 | 126.26 | 8.38 | 89.95 | 0.71 | 6 | 25 | 33 | 7 | 12 | 0.68 |
| 21 | 0.38 | 0.00 | 0.34 | 0.72 | 202.64 | 118.62 | 8.54 | 84.01 | 0.70 | 7 | 24 | 34 | 7 | 15 | 0.65 |
| 22 | 0.40 | 0.73 | 0.22 | 1.35 | 353.89 | 228.56 | 9.83 | 125.33 | 0.75 | 3 | 22 | 16 | 4 | 13 | 0.61 |
| 28 | 0.35 | 0.25 | 0.34 | 0.93 | 215.27 | 129.58 | 5.30 | 85.69 | 0.66 | 6 | 23 | 29 | 7 | 14 | 0.71 |
| 30 | 0.90 | 0.82 | 0.40 | 2.12 | 183.85 | 117.36 | 4.02 | 66.48 | 0.74 | 6 | 28 | 32 | 2 | 16 | 0.70 |
| 31 | 0.63 | 0.54 | 0.39 | 1.56 | 168.38 | 104.91 | 4.81 | 63.47 | 0.74 | 6 | 26 | 36 | 5 | 16 | 0.74 |
| 34 | 0.38 | 0.85 | 0.59 | 1.82 | 145.98 | 87.02 | 5.72 | 58.96 | 0.73 | 9 | 21 | 45 | 4 | 12 | 0.68 |
| 35 | 0.12 | 0.00 | 0.31 | 0.43 | 215.57 | 139.83 | 3.49 | 75.74 | 0.76 | 8 | 27 | 20 | 4 | 15 | 0.73 |
| 36 | 0.59 | 0.70 | 0.40 | 1.70 | 217.04 | 127.82 | 10.03 | 89.22 | 0.72 | 6 | 21 | 37 | 5 | 12 | 0.66 |
| 37 | 0.49 | 0.00 | 0.13 | 0.63 | 306.26 | 207.89 | 2.64 | 98.37 | 0.81 | 8 | 21 | 2 | 5 | 14 | 0.60 |
| 38 | 0.32 | 0.71 | 0.30 | 1.33 | 202.69 | 118.19 | 8.75 | 84.50 | 0.69 | 6 | 22 | 23 | 6 | 13 | 0.60 |
| 39 | 0.61 | 0.30 | 0.32 | 1.22 | 178.50 | 113.36 | 4.11 | 65.14 | 0.71 | 9 | 25 | 41 | 7 | 15 | 0.82 |
| 44 | 0.05 | 0.52 | 0.22 | 0.80 | 340.60 | 200.81 | 13.46 | 139.78 | 0.70 | 5 | 23 | 30 | 6 | 15 | 0.65 |
| 45 | 0.16 | 0.46 | 0.54 | 1.16 | 164.59 | 93.54 | 5.37 | 71.06 | 0.70 | 5 | 24 | 71 | 5 | 14 | 0.79 |
| 46 | 0.32 | 0.44 | 0.41 | 1.17 | 112.57 | 75.75 | 3.76 | 36.82 | 0.79 | 4 | 27 | 30 | 1 | 15 | 0.72 |
| 47 | 0.40 | 0.49 | 0.45 | 1.34 | 121.20 | 74.51 | 4.03 | 46.69 | 0.70 | 7 | 26 | 60 | 6 | 13 | 0.81 |
| 48 | 0.00 | 0.44 | 0.25 | 0.69 | 164.40 | 101.62 | 5.15 | 62.78 | 0.79 | 7 | 24 | 66 | 8 | 16 | 0.86 |
| 50 | 0.13 | 0.00 | 0.25 | 0.38 | 272.00 | 159.67 | 7.13 | 112.34 | 0.69 | 7 | 26 | 52 | 8 | 10 | 0.73 |
| 52 | 0.00 | 0.00 | 0.00 | 0.00 | 126.08 | 73.49 | 5.22 | 52.59 | 0.68 | 3 | 30 | 19 | 4 | 10 | 0.69 |
| 53 | 0.22 | 0.55 | 0.43 | 1.20 | 234.47 | 134.06 | 9.25 | 100.42 | 0.69 | 2 | 23 | 32 | 5 | 11 | 0.56 |
| 54 | 0.45 | 0.04 | 0.29 | 0.77 | 116.07 | 73.97 | 3.73 | 42.10 | 0.77 | 5 | 25 | 14 | 6 | 14 | 0.66 |
| 55 | 0.53 | 0.45 | 0.36 | 1.35 | 179.40 | 106.56 | 7.31 | 72.83 | 0.72 | 5 | 23 | 41 | 6 | 16 | 0.74 |
| 56 | 0.45 | 0.04 | 0.70 | 1.20 | 118.76 | 72.37 | 4.05 | 46.39 | 0.70 | 6 | 27 | 34 | 7 | 15 | 0.74 |
| 57 | 0.45 | 0.57 | 0.46 | 1.47 | 181.85 | 102.99 | 6.48 | 78.86 | 0.71 | 5 | 26 | 55 | 5 | 13 | 0.73 |
| 58 | 0.06 | 0.61 | 0.17 | 0.84 | 126.00 | 77.48 | 4.91 | 48.52 | 0.77 | 4 | 29 | 43 | 6 | 13 | 0.78 |
| 59 | 0.77 | 0.51 | 0.49 | 1.77 | 154.74 | 96.35 | 5.65 | 58.39 | 0.74 | 4 | 20 | 36 | 3 | 16 | 0.64 |
| 60 | 0.74 | 0.23 | 0.67 | 1.64 | 185.99 | 109.68 | 5.40 | 76.31 | 0.73 | 5 | 24 | 48 | 9 | 12 | 0.69 |
| 61 | 0.30 | 0.14 | 0.46 | 0.90 | 164.43 | 108.43 | 1.62 | 56.00 | 0.77 | 8 | 27 | 23 | 3 | 13 | 0.64 |
| 63 | 0.50 | 0.02 | 0.42 | 0.95 | 156.97 | 101.46 | 2.56 | 55.51 | 0.76 | 7 | 21 | 39 | 5 | 15 | 0.72 |
| 64 | 0.64 | 0.00 | 0.65 | 1.29 | 124.53 | 81.12 | 2.41 | 43.41 | 0.79 | 7 | 28 | 35 | 5 | 15 | 0.70 |
| 65 | 0.90 | 0.72 | 0.59 | 2.21 | 117.44 | 70.19 | 5.31 | 47.25 | 0.72 | 6 | 23 | 55 | 3 | 13 | 0.74 |
| 66 | 0.95 | 0.69 | 0.62 | 2.27 | 119.54 | 73.94 | 4.52 | 45.60 | 0.75 | 7 | 22 | 69 | 2 | 14 | 0.82 |
| 67 | 0.54 | 0.64 | 0.55 | 1.73 | 153.35 | 89.59 | 6.50 | 63.76 | 0.66 | 6 | 24 | 9 | 5 | 14 | 0.57 |
| 68 | 0.87 | 0.00 | 0.74 | 1.61 | 119.49 | 77.02 | 3.18 | 42.47 | 0.78 | 7 | 25 | 29 | 1 | 15 | 0.66 |
| 69 | 0.88 | 0.00 | 0.52 | 1.39 | 170.67 | 100.52 | 4.84 | 70.15 | 0.72 | 6 | 25 | 38 | 8 | 13 | 0.72 |
| 71 | 0.77 | 0.40 | 0.30 | 1.46 | 156.07 | 88.34 | 5.28 | 67.73 | 0.71 | 4 | 26 | 43 | 3 | 14 | 0.75 |
| 72 | 0.70 | 0.00 | 0.57 | 1.27 | 150.63 | 90.47 | 5.35 | 60.16 | 0.71 | 6 | 29 | 68 | 3 | 13 | 0.83 |
| 73 | 0.66 | 0.00 | 0.50 | 1.16 | 223.39 | 140.57 | 4.67 | 82.81 | 0.78 | 6 | 27 | 13 | 6 | 13 | 0.68 |
| 75 | 0.94 | 0.30 | 0.76 | 1.99 | 120.68 | 77.48 | 3.10 | 43.20 | 0.69 | 9 | 24 | 20 | 8 | 13 | 0.58 |
| 76 | 0.39 | 0.00 | 0.60 | 1.00 | 77.55 | 53.77 | 0.76 | 23.78 | 0.74 | 7 | 23 | 52 | 7 | 16 | 0.80 |
| 77 | 0.51 | 0.46 | 0.22 | 1.20 | 159.37 | 91.32 | 6.10 | 68.04 | 0.70 | 7 | 22 | 49 | 3 | 13 | 0.71 |
| 78 | 0.00 | 0.00 | 0.35 | 0.35 | 227.11 | 147.67 | 4.35 | 79.44 | 0.72 | 6 | 21 | 47 | 7 | 14 | 0.70 |
| 79 | 0.17 | 0.00 | 0.35 | 0.52 | 181.41 | 104.70 | 7.00 | 76.71 | 0.69 | 4 | 27 | 36 | 8 | 16 | 0.69 |
| 83 | 0.59 | 0.77 | 0.54 | 1.90 | 214.31 | 128.56 | 7.99 | 85.75 | 0.74 | 4 | 25 | 36 | 6 | 13 | 0.63 |
| 84 | 0.92 | 0.45 | 0.41 | 1.77 | 204.40 | 120.32 | 8.72 | 84.09 | 0.70 | 8 | 30 | 18 | 7 | 15 | 0.67 |
| 85 | 0.84 | 0.20 | 0.51 | 1.56 | 172.86 | 100.42 | 6.77 | 72.44 | 0.69 | 6 | 27 | 8 | 5 | 12 | 0.56 |
| 86 | 0.68 | 0.87 | 0.44 | 1.99 | 177.32 | 104.79 | 7.84 | 72.52 | 0.68 | 5 | 31 | 37 | 3 | 13 | 0.68 |
| 87 | 0.75 | 0.80 | 0.57 | 2.12 | 187.32 | 110.54 | 7.93 | 76.78 | 0.72 | 4 | 22 | 38 | 4 | 15 | 0.64 |
| 89 | 0.87 | 0.34 | 0.41 | 1.62 | 304.11 | 177.48 | 8.80 | 126.63 | 0.72 | 6 | 26 | 40 | 7 | 12 | 0.69 |
| 91 | 0.55 | 0.41 | 0.49 | 1.46 | 194.18 | 116.88 | 4.76 | 77.29 | 0.70 | 5 | 20 | 23 | 7 | 15 | 0.60 |
| 93 | 0.00 | 0.32 | 0.00 | 0.32 | 205.74 | 124.41 | 5.45 | 81.33 | 0.68 | 7 | 21 | 50 | 8 | 16 | 0.78 |
| 94 | 0.67 | 0.40 | 0.77 | 1.85 | 88.68 | 56.92 | 3.14 | 31.75 | 0.75 | 7 | 19 | 28 | 7 | 11 | 0.61 |
| 96 | 0.17 | 0.00 | 0.35 | 0.52 | 159.25 | 105.67 | 3.09 | 53.58 | 0.76 | 7 | 26 | 17 | 6 | 13 | 0.56 |
| 98 | 0.10 | 0.00 | 0.29 | 0.39 | 183.57 | 107.30 | 8.14 | 76.27 | 0.68 | 5 | 22 | 16 | 5 | 13 | 0.59 |
| 101 | 0.14 | 0.00 | 0.42 | 0.56 | 179.28 | 104.29 | 6.23 | 75.00 | 0.72 | 7 | 26 | 13 | 6 | 16 | 0.62 |
| 102 | 0.40 | 0.67 | 0.46 | 1.52 | 222.71 | 131.67 | 9.48 | 91.04 | 0.70 | 5 | 25 | 21 | 4 | 12 | 0.66 |
| 103 | 0.21 | 0.70 | 0.45 | 1.36 | 130.54 | 77.33 | 6.10 | 53.21 | 0.73 | 7 | 29 | 51 | 4 | 12 | 0.78 |
| 104 | 0.07 | 0.41 | 0.42 | 0.90 | 108.52 | 73.07 | 3.22 | 35.45 | 0.82 | 9 | 21 | 25 | 7 | 17 | 0.60 |
| 105 | 0.26 | 0.55 | 0.28 | 1.09 | 187.94 | 108.47 | 7.14 | 79.48 | 0.72 | 5 | 24 | 58 | 6 | 16 | 0.77 |
| 106 | 0.48 | 0.06 | 0.55 | 1.09 | 147.58 | 99.01 | 2.14 | 48.57 | 0.71 | 6 | 25 | 18 | 4 | 13 | 0.54 |
| 107 | 0.39 | 0.07 | 0.57 | 1.04 | 165.03 | 102.98 | 4.97 | 62.05 | 0.72 | 6 | 17 | 58 | 5 | 15 | 0.73 |
| 108 | 0.73 | 0.00 | 0.72 | 1.45 | 98.48 | 61.85 | 2.62 | 36.63 | 0.70 | 6 | 19 | 15 | 3 | 13 | 0.60 |
| 109 | 0.84 | 0.00 | 0.68 | 1.53 | 132.50 | 79.81 | 4.19 | 52.69 | 0.73 | 7 | 23 | 28 | 8 | 13 | 0.71 |
| 110 | 0.73 | 0.88 | 0.42 | 2.03 | 172.03 | 101.10 | 7.70 | 70.93 | 0.73 | 4 | 25 | 38 | 5 | 13 | 0.72 |
| 111 | 0.21 | 0.27 | 0.62 | 1.09 | 79.11 | 48.21 | 2.77 | 30.90 | 0.72 | 7 | 20 | 32 | 7 | 13 | 0.66 |
| 113 | 0.35 | 0.00 | 0.55 | 0.90 | 161.28 | 96.78 | 7.84 | 64.50 | 0.66 | 6 | 25 | 36 | 3 | 15 | 0.72 |
| 114 | 0.47 | 0.46 | 0.31 | 1.25 | 261.59 | 159.84 | 6.57 | 101.74 | 0.70 | 7 | 23 | 47 | 7 | 16 | 0.83 |
| 117 | 0.59 | 0.70 | 0.63 | 1.92 | 187.28 | 113.20 | 8.67 | 74.08 | 0.74 | 5 | 25 | 29 | 7 | 12 | 0.62 |
| 119 | 0.38 | 0.00 | 0.14 | 0.52 | 209.73 | 131.95 | 4.06 | 77.79 | 0.73 | 6 | 28 | 44 | 8 | 13 | 0.74 |
| 121 | 0.85 | 0.26 | 0.61 | 1.72 | 180.96 | 123.76 | 2.63 | 57.20 | 0.78 | 7 | 27 | 35 | 5 | 14 | 0.74 |
| 122 | 0.09 | 0.34 | 0.47 | 0.90 | 192.98 | 127.14 | 2.17 | 65.84 | 0.72 | 8 | 21 | 14 | 4 | 16 | 0.57 |
| 123 | 0.16 | 0.00 | 0.40 | 0.56 | 132.55 | 79.75 | 4.03 | 52.80 | 0.61 | 8 | 18 | 30 | 7 | 16 | 0.66 |
| 124 | 0.53 | 0.63 | 0.47 | 1.63 | 137.72 | 81.78 | 6.18 | 55.94 | 0.68 | 3 | 22 | 37 | 6 | 13 | 0.69 |
| 125 | 0.44 | 0.39 | 0.30 | 1.12 | 135.36 | 84.15 | 4.27 | 51.21 | 0.76 | 5 | 24 | 48 | 6 | 14 | 0.74 |
| 127 | 0.83 | 0.16 | 0.43 | 1.42 | 217.36 | 129.98 | 7.02 | 87.38 | 0.72 | 5 | 24 | 15 | 4 | 16 | 0.62 |
| 128 | 0.52 | 0.78 | 0.35 | 1.65 | 217.48 | 129.96 | 8.57 | 87.52 | 0.71 | 4 | 23 | 43 | 8 | 12 | 0.67 |
| 129 | 0.77 | 0.04 | 0.40 | 1.21 | 230.09 | 153.29 | 3.59 | 76.79 | 0.78 | 5 | 26 | 38 | 8 | 13 | 0.69 |
| 130 | 0.49 | 0.31 | 0.39 | 1.19 | 221.53 | 135.46 | 6.46 | 86.07 | 0.74 | 8 | 25 | 28 | 8 | 13 | 0.72 |
| 131 | 0.82 | 0.00 | 0.26 | 1.08 | 241.84 | 138.06 | 9.69 | 103.78 | 0.69 | 6 | 23 | 39 | 7 | 17 | 0.73 |
| 132 | 0.35 | 0.38 | 0.44 | 1.18 | 185.70 | 105.92 | 7.05 | 79.78 | 0.68 | 6 | 26 | 8 | 5 | 14 | 0.51 |
| 133 | 0.08 | 0.00 | 0.44 | 0.53 | 239.70 | 148.95 | 5.13 | 90.76 | 0.73 | 4 | 24 | 31 | 6 | 16 | 0.58 |
| 134 | 0.75 | 0.21 | 0.41 | 1.37 | 205.54 | 119.56 | 7.55 | 85.98 | 0.72 | 5 | 21 | 12 | 4 | 15 | 0.56 |
| 135 | 0.21 | 0.63 | 0.26 | 1.10 | 236.23 | 142.37 | 10.32 | 93.86 | 0.73 | 5 | 24 | 12 | 5 | 14 | 0.60 |
| 136 | 0.53 | 0.42 | 0.54 | 1.50 | 213.02 | 123.99 | 8.29 | 89.03 | 0.70 | 4 | 21 | 23 | 6 | 12 | 0.54 |
| 137 | 0.53 | 0.54 | 0.38 | 1.44 | 184.45 | 104.99 | 6.33 | 79.46 | 0.72 | 7 | 25 | 41 | 4 | 16 | 0.68 |
| 138 | 0.19 | 0.72 | 0.31 | 1.22 | 194.23 | 112.16 | 7.83 | 82.06 | 0.71 | 6 | 17 | 20 | 4 | 12 | 0.58 |
| 140 | 0.58 | 0.51 | 0.38 | 1.47 | 204.63 | 122.22 | 8.24 | 82.42 | 0.68 | 6 | 21 | 13 | 3 | 15 | 0.55 |
| 141 | 0.29 | 0.28 | 0.37 | 0.94 | 154.02 | 87.16 | 5.20 | 66.86 | 0.67 | 7 | 24 | 10 | 2 | 12 | 0.60 |
| 148 | 0.42 | 0.38 | 0.46 | 1.26 | 163.14 | 99.66 | 6.19 | 63.48 | 0.71 | 7 | 22 | 28 | 5 | 17 | 0.71 |
| 151 | 0.37 | 0.43 | 0.54 | 1.34 | 183.17 | 111.85 | 6.29 | 71.32 | 0.70 | 6 | 18 | 42 | 3 | 16 | 0.62 |
| 152 | 0.27 | 0.50 | 0.14 | 0.91 | 388.42 | 228.51 | 15.67 | 159.91 | 0.71 | 6 | 21 | 32 | 6 | 14 | 0.63 |
| 153 | 0.86 | 0.89 | 0.31 | 2.05 | 280.11 | 160.65 | 11.39 | 119.45 | 0.68 | 6 | 19 | 26 | 6 | 13 | 0.62 |
| 156 | 0.71 | 0.55 | 0.42 | 1.68 | 162.44 | 97.87 | 6.54 | 64.57 | 0.66 | 6 | 18 | 29 | 4 | 14 | 0.62 |
| 159 | 0.93 | 0.52 | 0.33 | 1.77 | 188.18 | 110.60 | 6.58 | 77.58 | 0.72 | 3 | 20 | 31 | 1 | 14 | 0.52 |
| 160 | 0.13 | 0.17 | 0.41 | 0.71 | 202.36 | 124.97 | 5.74 | 77.39 | 0.73 | 6 | 18 | 17 | 7 | 15 | 0.56 |
| 161 | 0.00 | 0.61 | 0.21 | 0.82 | 260.56 | 155.77 | 9.91 | 104.80 | 0.73 | 7 | 20 | 43 | 4 | 15 | 0.76 |
| 162 | 0.57 | 0.50 | 0.39 | 1.45 | 137.53 | 81.60 | 6.40 | 55.93 | 0.69 | 6 | 15 | 10 | 6 | 12 | 0.50 |
| 163 | 0.52 | 0.49 | 0.28 | 1.29 | 198.46 | 117.72 | 7.48 | 80.73 | 0.70 | 5 | 20 | 34 | 2 | 14 | 0.65 |
| 164 | 0.54 | 0.78 | 0.40 | 1.71 | 197.38 | 117.26 | 9.21 | 80.13 | 0.71 | 6 | 17 | 11 | 4 | 13 | 0.51 |
| 165 | 0.40 | 0.42 | 0.48 | 1.29 | 156.98 | 98.49 | 5.77 | 58.49 | 0.75 | 4 | 23 | 44 | 5 | 17 | 0.71 |
| 167 | 0.70 | 0.82 | 0.36 | 1.88 | 178.95 | 109.81 | 8.75 | 69.14 | 0.74 | 5 | 18 | 47 | 2 | 16 | 0.76 |
| 168 | 0.25 | 0.65 | 0.43 | 1.32 | 175.09 | 101.59 | 7.53 | 73.50 | 0.70 | 6 | 17 | 34 | 3 | 13 | 0.61 |
| 171 | 0.36 | 0.58 | 0.57 | 1.51 | 210.22 | 131.98 | 7.18 | 78.24 | 0.75 | 5 | 20 | 14 | 5 | 14 | 0.59 |
| 172 | 0.49 | 0.67 | 0.61 | 1.78 | 178.69 | 108.86 | 6.80 | 69.82 | 0.71 | 5 | 23 | 40 | 6 | 13 | 0.69 |
| 173 | 0.60 | 0.65 | 0.53 | 1.79 | 150.28 | 89.06 | 6.58 | 61.23 | 0.71 | 5 | 20 | 10 | 3 | 15 | 0.57 |
| 176 | 0.62 | 0.55 | 0.30 | 1.47 | 257.31 | 148.51 | 8.83 | 108.80 | 0.72 | 5 | 15 | 30 | 4 | 13 | 0.59 |
| 178 | 0.00 | 0.00 | 0.00 | 0.00 | 199.96 | 136.73 | 2.72 | 63.23 | 0.71 | 6 | 19 | 12 | 6 | 12 | 0.54 |
| 179 | 0.32 | 0.88 | 0.29 | 1.49 | 198.83 | 118.54 | 8.99 | 80.30 | 0.68 | 3 | 22 | 46 | 5 | 11 | 0.58 |
| 180 | 0.22 | 0.65 | 0.43 | 1.30 | 164.34 | 99.20 | 6.87 | 65.15 | 0.69 | 6 | 18 | 9 | 7 | 15 | 0.56 |
| 181 | 0.68 | 0.45 | 0.41 | 1.54 | 215.48 | 130.48 | 8.81 | 85.00 | 0.70 | 8 | 25 | 40 | 5 | 13 | 0.71 |
| 182 | 0.06 | 0.00 | 0.39 | 0.45 | 214.44 | 134.14 | 4.33 | 80.30 | 0.75 | 4 | 26 | 16 | 7 | 13 | 0.62 |
| 183 | 0.40 | 0.66 | 0.64 | 1.70 | 104.81 | 61.85 | 4.75 | 42.97 | 0.71 | 6 | 26 | 30 | 4 | 13 | 0.67 |
| 184 | 0.04 | 0.52 | 0.51 | 1.07 | 172.32 | 101.50 | 6.41 | 70.82 | 0.69 | 4 | 21 | 8 | 7 | 14 | 0.53 |
| 185 | 0.59 | 0.88 | 0.23 | 1.69 | 209.10 | 125.29 | 9.58 | 83.80 | 0.69 | 5 | 19 | 43 | 4 | 13 | 0.63 |
| 186 | 0.38 | 0.27 | 0.28 | 0.94 | 130.48 | 87.11 | 3.55 | 43.37 | 0.72 | 5 | 21 | 40 | 7 | 14 | 0.64 |
| 187 | 0.59 | 0.44 | 0.41 | 1.44 | 234.57 | 136.15 | 7.68 | 98.42 | 0.67 | 6 | 26 | 46 | 3 | 16 | 0.77 |
| 188 | 0.47 | 0.66 | 0.50 | 1.63 | 213.86 | 123.89 | 8.56 | 89.97 | 0.71 | 5 | 23 | 39 | 4 | 14 | 0.70 |

Table S2 – Results of Pearson’s test for correlations between continuous predictors (-1 = perfect negative correlation, 0 = no correlation, 1= perfect positive correlation).

|  | ldwcut | Inonat | Iharv | ForMI | Total carbon | Above ground carbon | Foliage carbon | Root carbon | NDVI |
| --- | --- | --- | --- | --- | --- | --- | --- | --- | --- |
| ldwcut | 1 |  |  |  |  |  |  |  |  |
| Inonat | 0.19 | 1 |  |  |  |  |  |  |  |
| Iharv | 0.49 | 0.00 | 1 |  |  |  |  |  |  |
| ForMI | 0.81 | 0.66 | 0.59 | 1 |  |  |  |  |  |
| Total carbon | -0.03 | 0.16 | -0.34 | -0.04 | 1 |  |  |  |  |
| Aboveground carbon | -0.04 | 0.10 | -0.34 | -0.08 | 0.99 | 1 |  |  |  |
| Foliage carbon | 0.06 | 0.56 | -0.21 | 0.28 | 0.66 | 0.56 | 1 |  |  |
| Root carbon | -0.01 | 0.25 | -0.33 | 0.02 | 0.97 | 0.92 | 0.78 | 1 |  |
| NDVI | 0.13 | -0.17 | 0.19 | 0.04 | -0.09 | 0.00 | -0.37 | -0.23 | 1 |

Table S3 – Pseudo R² values of the randomForest models where FCN refers to the ForMI, total carbon and NDVI as predictors, ForMI components refer to the ratio of artificial vs. natural deadwood (Idwcut), share of non-native tree species (Inonat) and ratio of harvested volume (Iharv) as predictors and carbon pools include aboveground, foliage and root carbon as predictors.

| Model | Pseudo R² |
| --- | --- |
| FCN |  |
| *Pooled richness* | -0.16 |
| *Plants* | -0.14 |
| *Insects* | -0.03 |
| *Bats* | -0.16 |
| *Birds* | -0.15 |
| *TreMs* | -0.06 |
| ForMI components |  |
| *Pooled richness* | -0.06 |
| *Plants* | -0.17 |
| *Insects* | -0.09 |
| *Bats* | -0.23 |
| *Birds* | -0.24 |
| *TreMs* | -0.10 |
| Carbon components |  |
| *Pooled richness* | -0.03 |
| *Plants* | -0.05 |
| *Insects* | -0.14 |
| *Bats* | -0.22 |
| *Birds* | -0.18 |
| *TreMs* | -0.05 |

Table S4 – Negative and positive change points of the threshold indicator taxa analysis for all predictors (environmental gradients) per taxonomic group. The “share of interval” is the range of the 5-95% change point quantiles in comparison to the maximum range of the predictor. The bold letters indicate where the share is below 15% and thus considered a threshold.

| Taxonomic groups per predictor | Negative change point (NCP) | Positive change point (PCP) | Min of predictor | Max of predictor | Share interval NCP | Share interval PCP |
| --- | --- | --- | --- | --- | --- | --- |
| ForMI |  |  |  |  |  |  |
| - *TreMs* | 0.75 | 1.02 | 0 | 2.4 | **12%** | 42% |
| - *Birds* | 0.69 | 1.89 | 0 | 2.4 | 34% | 22% |
| - *Bats* | 1.00 | 1.86 | 0 | 2.4 | 30% | 61% |
| - *Insects* | 0.36 | 0.48 | 0 | 2.4 | 31% | 36% |
| - *Plants* | 0.59 | 1.80 | 0 | 2.4 | 23% | 31% |
| - *Pooled richness* | 1.00 | 1.86 | 0 | 2.4 | 26% | **15%** |
| Total carbon (MgC/ha) | |  |  |  |  |  |
| - *TreMs* | 116.47 | 176.20 | 48.5 | 388.4 | **12%** | 22% |
| - *Birds* | 177.91 | 116.47 | 48.5 | 388.4 | 41% | 32% |
| - *Bats* | 110.54 | NA | 48.5 | 388.4 | 21% | NA |
| - *Insects* | 209.41 | 213.26 | 48.5 | 388.4 | 26% | 52% |
| - *Plants* | 166.71 | 250.25 | 48.5 | 388.4 | 18% | 28% |
| - *Pooled richness* | 172.17 | 164.81 | 48.5 | 388.4 | 17% | 36% |
| NDVI |  |  |  |  |  |  |
| - *TreMs* | 0.68 | 0.77 | 0.6 | 0.8 | 38% | 34% |
| - *Birds* | 0.69 | 0.74 | 0.6 | 0.8 | **12%** | 28% |
| - *Bats* | 0.68 | 0.74 | 0.6 | 0.8 | 31% | 26% |
| - *Insects* | 0.70 | 0.73 | 0.6 | 0.8 | 38% | 29% |
| - *Plants* | 0.68 | 0.74 | 0.6 | 0.8 | 25% | 32% |
| - *Pooled richness* | 0.69 | 0.73 | 0.6 | 0.8 | 17% | 29% |
| Idwcut |  |  |  |  |  |  |
| - *TreMs* | 0.19 | 0.71 | 0 | 1 | 24% | 31% |
| - *Birds* | 0.07 | 0.64 | 0 | 1 | 45% | 42% |
| - *Bats* | NA | 0.58 | 0 | 1 | NA | 78% |
| - *Insects* | 0.17 | 0.00 | 0 | 1 | 50% | 90% |
| - *Plants* | 0.49 | 0.85 | 0 | 1 | 53% | 31% |
| - *Pooled richness* | 0.43 | 0.62 | 0 | 1 | 53% | 41% |
| Inonat |  |  |  |  |  |  |
| - *TreMs* | 0.34 | 0.29 | 0 | 0.9 | 47% | 45% |
| - *Birds* | 0.30 | 0.54 | 0 | 0.9 | 58% | 55% |
| - *Bats* | 0.53 | NA | 0 | 0.9 | 61% | NA |
| - *Insects* | 0.52 | 0.41 | 0 | 0.9 | 42% | 52% |
| - *Plants* | 0.55 | 0.55 | 0 | 0.9 | 62% | 31% |
| - *Pooled richness* | 0.17 | 0.55 | 0 | 0.9 | 60% | 39% |
| Iharv |  |  |  |  |  |  |
| - *TreMs* | 0.22 | 0.64 | 0 | 0.8 | 23% | 62% |
| - *Birds* | 0.28 | 0.44 | 0 | 0.8 | 74% | 52% |
| - *Bats* | 0.14 | 0.44 | 0 | 0.8 | 20% | 28% |
| - *Insects* | 0.22 | 0.14 | 0 | 0.8 | 64% | 69% |
| - *Plants* | 0.20 | 0.57 | 0 | 0.8 | 37% | **12%** |
| - *Pooled richness* | 0.20 | 0.56 | 0 | 0.8 | 29% | **10%** |
| Aboveground carbon (MgC/ha) | | |  |  |  |  |
| - *TreMs* | 60.21 | 136.30 | 28.9 | 228.6 | **14%** | **15%** |
| - *Birds* | 110.18 | 60.21 | 28.9 | 228.6 | 40% | 34% |
| - *Bats* | 79.78 | 98.18 | 28.9 | 228.6 | 24% | 20% |
| - *Insects* | 104.95 | 125.78 | 28.9 | 228.6 | 21% | 43% |
| - *Plants* | 103.64 | 148.73 | 28.9 | 228.6 | **8%** | 17% |
| - *Pooled richness* | 109.75 | 126.70 | 28.9 | 228.6 | **8%** | 18% |
| Foliage carbon(MgC/ha) | |  |  |  |  |  |
| - *TreMs* | 3.74 | 7.76 | 0.8 | 15.7 | 16% | 32% |
| - *Birds* | 5.43 | 5.69 | 0.8 | 15.7 | 18% | 23% |
| - *Bats* | 3.53 | NA | 0.8 | 15.7 | 22% | NA |
| - *Insects* | 6.10 | 5.69 | 0.8 | 15.7 | 17% | 17% |
| - *Plants* | 5.69 | 7.70 | 0.8 | 15.7 | 32% | 17% |
| - *Pooled richness* | 5.43 | 5.69 | 0.8 | 15.7 | **14%** | 19% |
| Root carbon(MgC/ha) | |  |  |  |  |  |
| - *TreMs* | 42.29 | 84.75 | 19.6 | 159.9 | **9%** | 20% |
| - *Birds* | 61.64 | 52.75 | 19.6 | 159.9 | 41% | 28% |
| - *Bats* | 56.60 | NA | 19.6 | 159.9 | 23% | NA |
| - *Insects* | NA | 85.34 | 19.6 | 159.9 | NA | 23% |
| - *Plants* | 48.54 | 98.40 | 19.6 | 159.9 | **15%** | 23% |
| - *Pooled* | 58.44 | 68.44 | 19.6 | 159.9 | 18% | 33% |
